# Chlorine as a geobarometer for alkaline magmas: Evidence from a systematic study of the eruptions of Mount Somma-Vesuvius

**DOI:** 10.1038/srep21726

**Published:** 2016-02-18

**Authors:** H. Balcone-Boissard, G. Boudon, R. Cioni, J. D. Webster, G. Zdanowicz, G. Orsi, L. Civetta

**Affiliations:** 1 Sorbonne Universités, UPMC Univ. Paris 06, CNRS, Institut des Sciences de la Terre de Paris (iSTeP), 4 place Jussieu 75005 Paris, France; 2 Institut de Physique du Globe de Paris, Sorbonne Paris Cité, Univ Paris Diderot, CNRS, F-75005 Paris, France; 3 Dip.to Scienze della Terra, Universita’ degli Studi di Firenze, Via La Pira 4, 50121 Firenze, Italy; 4 Department of Earth and Planetary Sciences, American Museum of Natural History, Central Park West at 79th St., NY, NY 10024-5192 USA; 5 Dipartimento di Scienze della Terra, dell’Ambiente e delle Risorse, Università degli Studi di Napoli Federico II, Largo S. Marcellino 10, 80138 Napoli, Italy; 6 Dipartimento di Fisica “E. R. Caianiello” Università degli Studi di Salerno, Via Giovanni Paolo II 132, 84084 Fisciano, Salerno, Italy; 7 Istituto Nazionale di Geofisica e Vulcanologia, Sezione di Palermo, Via Ugo La Malfa 153, 90146 Palermo, Italy

## Abstract

Defining the magma storage conditions of a volcanic system is a major goal in modern volcanology due to its direct implications for the style of a possible eruption, and thus on the associated risk of any crisis and the necessary management and mitigation strategies. Below 200 MPa and at equivalent depths, the strongly non-ideal behaviour of the H-C-O-S-Cl-F system in the silicate melt causes unmixing of the fluid phase to form an H_2_O-rich vapour and a hydrosaline phase in equilibrium with the silicate melt, both responsible for buffering the chlorine (Cl) concentration. Following this equilibrium, the Cl concentration in melts may be used as a geobarometer for alkaline magmas. Systematic application of this method to the main explosive eruptions of Mount Somma-Vesuvius highlights two main magma ponding zones, at ~180–200 and ~100 MPa. At these pressures, the maximum pre-eruptive H_2_O contents for the different magma compositions can be estimated; the results obtained, largely in agreement with the current literature, therefore confirm the validity of the method. The Cl geobarometer may help scientists to define the variation of the magmatic reservoir location through time and thus provide strong constraints on pre-eruptive conditions, which are of utmost importance for volcanic crisis management.

Experimental studies on the solubility of volatiles (H_2_O, CO_2_, S, Cl, F) in fluid-saturated silicate melts show that Cl preferentially partitions into aqueous fluids rather than silicate melts^1^,^2^,^3^,^4^,^5^. The NaCl-H_2_O system is representative of magmatic fluids and is characterized by fluid immiscibility under a wide range of P-T conditions, and NaCl contents (P < 200 MPa, T < 1000 °C^6^,^7^,^8^,^9^; Fig. 1), and NaCl-H_2_O fluids in the presence of silicate melt similarly display immiscibility. The P-T conditions noted above are commonly encountered where magmas are evolving in shallow reservoirs, and the associated immiscibility strongly affects the evolution of magmatic melts and fluids. Numerous experimental studies on Cl solubility have highlighted its saturation conditions in silicate melts^3^,^4^,^6^,^7^,^8^,^9^,^10^,^11^,^12^,^13^,^14^ especially in alkaline magmas; however, few studies with natural samples^15^ are presently available. Experimental results shed light on the importance of considering the more general melt-H-C-O-S-Cl-F pseudo-system, in order to describe the behaviour of the fluid phase and its relationships with the silicate melt. Recent experimental data^16^,^17^ point out the possible complex interactions among volatiles in the fluid phase, depending on mixing processes and volatile speciation, which in turn influence volatile solubility in a silicate melt. Even if H_2_O is the dominant species and plays a prominent role in magma behaviour, mainly through decompressional exsolution^18^, CO_2_^19^, Cl^4^,^5^, S^16^,^20^,^21^, and, to a lesser extent, F^16^ also play a significant role in determining the conditions of fluid saturation of a silicate melt. The solubility of Cl in silicate melt largely depends upon melt composition^2^,^3^,^4^,^22^,^23^,^24^ (being higher in alkali-rich magmas)^13^, pressure^11^ and the co-occurrence of other volatile species, such as S^20^,^21^. Temperature has little effect on Cl solubility^4^. In H_2_O-bearing systems, the Cl concentration in the exsolved vapour may increase with increasing Cl concentration in the silicate melt. Yet, these systems exhibit strongly non-ideal mixing properties between H_2_O, CO_2_, and Cl. If, depending on P-T conditions, the crystallizing magma is at solvus or sub-solvus conditions (in the system H_2_O-NaCl), the exsolved fluid phase unmixes to form a vapour and a brine, and so what was a pseudo-binary system becomes pseudo-ternary (Fig. 1). In such cases, the Cl partitioning behaviour is complex, as it is ruled by the coexistence of three phases in the volcanic system (vapour, silicate melt and brine). Because of this three-way partitioning, Cl exerts a strong control on fluid phase equilibria at the magmatic P-T conditions^8^,^25^,^26^,^27^, with the stability of the vapour and brine at sub-solvus conditions dependent on Cl activity in the fluids^14^,^27^. In such a sub-critical domain at constant P and T, the Cl concentration in the silicate melt is invariant as the composition of both vapour phase and brine is fixed, due to Gibb’s phase rule. This non-ideal behaviour of the fluid is expressed in the magmatic system by a buffered value of the Cl concentration in the silicate melt. The precise Cl buffer value varies with pressure: it increases at low pressure, as the immiscibility gap of the H_2_O-salt system becomes wider, and decreases at higher pressure (Fig. 1). At magmatic T and shallow crustal P, the pseudo-ternary H_2_O-NaCl-melt system predicts the presence of both vapour and brine, even for low NaCl contents^17^. During magma differentiation at hyper-solvus conditions, an increasing activity of Cl in the fluid may be ultimately responsible for the increase of the non-ideality of the mixing process among volatiles. Conversely, during differentiation at sub-solvus conditions, H_2_O and Cl concentrations in the melt, vapour and brine will be fixed, while the vapour/brine ratio decreases with increasing Cl in the system^17^ (Fig. 2). The solubility relationships may be integrated to interpret the Cl content in terms of the pressure conditions at which the silicate melt is stored.

The aim of this paper is to demonstrate the power of Cl as a geobarometer for alkaline magmas, once the buffering effect of its concentration has been identified. The case study we use is based on the detailed study of material from several explosive eruptions, of variable magnitude, of Mount Somma-Vesuvius (MSV). For the purposes of geobarometry, we propose to use the systematic Cl buffering effect on the silicate melt to estimate the pressure, and hence depth, of the magmatic reservoir feeding a given eruption from solubility data (Fig. 3). The existence of such a buffering effect for the magmas feeding the studied eruptions has been systematically detected on the basis of the Cl content in the residual matrix glass. The Cl geobarometer may be used for a single eruption or, if sampling and chemistry permits, to reconstruct the behaviour of a volcanic system through time, as we do here for MSV. In addition, the maximum pre-eruptive H_2_O content dissolved in a magmatic melt can be estimated using the corresponding experimental H_2_O solubility law^28^,^29^,^30^,^31^, based on the values of pressure of the magma storage derived from the Cl buffer (Fig. 4). As for Cl, the H_2_O solubility model should take into account the influence of other volatiles, such as CO_2_^17^,^32^,^33^, as proposed in Cl-H_2_O solubility models^17^.

## Terminology

Hereafter, we shall take *silicate melt* to refer to a molten silicate magmatic body that evolves by fractional crystallization in a reservoir within the crust. *Fluid* is a general term to mean a multicomponent, non-crystalline phase. In volcanic environments the volatile components exsolve as a *vapour phase*, which is a low-density fluid phase dominated by H_2_O and/or CO_2_ with more or less S species (SO_2_, H_2_S), and a *brine,* or *hypersaline liquid*, being a halogen (Cl and F as halide) species-rich, water-bearing fluid of magmatic origin. Silicate melt may be in equilibrium with either a vapour or a brine, or both. *Volatiles* is a general term referring to all species that may strongly partition in favour of a fluid phase. The *solubility* of a given volatile species in a silicate melt is the amount of that species dissolvable into that melt, at given P-T conditions, when the melt is saturated in either vapour (for H_2_O solubility), or brine (for Cl solubility) or both (for both H_2_O and Cl solubility). It also depends upon the mechanism through which it is incorporated in the silicate network, i.e. its speciation in the melt phase.

## How to use Cl as a geobarometer?

To infer magma storage conditions from Cl concentrations detected in silicate glass, it is necessary to take into account (1) the influence of the silicate melt composition and (2) the effect of the fluid composition on Cl behaviour.

### The composition of the melt

The influence of the evolutionary path of a silicate melt on Cl solubility was first suspected on the basis of the results of experimental studies that shed light on the role of the (Na + K)/Al and Al/Si molar ratios^3^,^11^, and the alkali^7^,^9^ and CaO contents^23^. Modelling of the predicted Cl content in silicate melt, extrapolated from the results of solubility experiments, demonstrates the major role played by the silicate melt composition on Cl solubility^13^. Modelling also highlights the influence of each major cation on Cl solubility. Such multi-component considerations allow us to take into account the significant changes in Cl behaviour resulting from fractional crystallisation of a silicate melt, even for an apparently subtle variation of its composition. During crystallisation, the Cl concentration in a fluid-free silicate melt increases as it behaves as an incompatible element (Fig. 2). Simultaneous decreases in Mg, Ca, Fe, and Al content may be responsible for the direct exsolution of a brine^17^. Thus, melt differentiation with accompanying increase of the Cl concentration in residual melt, leads to brine exsolution, with or without vapour, which may be seen as a particular form of second boiling^17^.

### The effect of other volatile species

**Figure 1:**
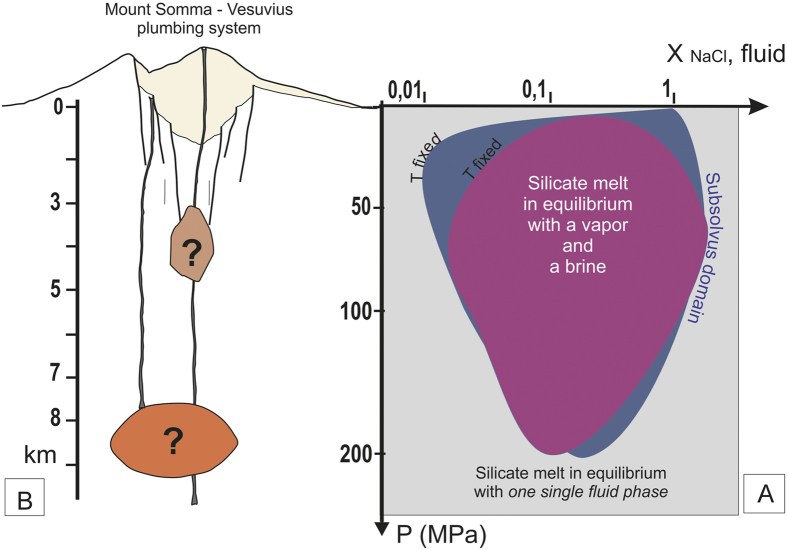
Link between Cl behaviour and the geometry of a plumbing system. (**A**) the H_2_O-NaCl-silicate melt pseudo-system, redrawn from phase equilibrium data^6^,^7^. Blue and purple domains: the silicate melt is in equilibrium with fluids, made of a vapour (H_2_O and/or CO_2_ and or S-rich) and a brine; the two colours correspond to two different melt temperatures. Within this subsolvus domain, drawn here for a given temperature, the Cl concentration in the silicate melt is buffered. Grey domain: the domain at higher P (T is fixed) than the solvus; the silicate melt is in equilibrium with a single fluid phase (see text for details). (**B**) Cross section of a plumbing system. Question marks: the position of the shallow crustal reservoir(s) will be constrained by Cl concentration in the silicate melt.

The behaviour of the volatiles in a fluid composed of only one phase (vapour or brine), differs from that in a fluid composed of coexisting immiscible vapour and brine. At sub-solvus conditions, when applied to the melt composition of interest, Cl modelling can provide the T-P conditions that best reproduce the Cl content measured in the same glass. Importantly, Cl solubility is also strongly affected by the presence of other volatile species, such as S. In fact, Cl solubility decreases by up to 30% in S-rich silicate melts^13^,^16^,^20^,^21^,^34^, so that Cl is observed to be most abundant in S-poor melts^16^. As S substantially reduces the solubility of Cl in silicate melts under oxidizing conditions^17^, and given the comparatively high oxygen fugacity and S contents of MSV magmas, the S effect in these magmas is likely to be significant. For this reason, the S effect has to be taken into account during Cl solubility modelling. The role played by F in terms of Cl solubility is less clear, although Cl solubility likely increases with increasing F^13^. Variation of the characteristics (i.e., compositions and phase relations) of the fluids due to the addition of other volatile components may also affect the Cl concentration in silicate melts (Fig. 1). For instance, an increase in CO_2_ will lead to an increase in the non-ideality of the volatile mixing processes^16^. In the presence of both vapour and brine, CO_2_ expands the stability field of the two fluids^35^, whereas S reduces this field, thus favouring mixing^16^. Such competing effects need to therefore be modelled to accurately determine Cl solubility.

### Solubility and equilibrium

**Figure 2:**
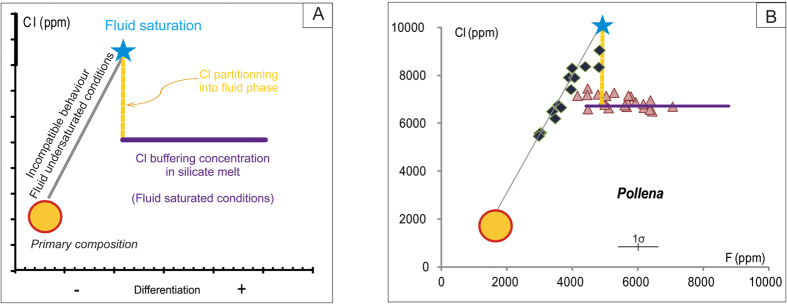
Cl behaviour and evidence of the buffering effect in silicate melt (melt inclusion and residual matrix glass composition). (**A**) Theoretical Cl behaviour. From the initial melt composition at a given Cl content (orange domain), melt differentiation under fluid-undersaturated conditions explains the Cl increase that is linearly correlated with the differentiation index (such as CaO or F) through the behaviour of an incompatible non-volatile component (solid grey line). When fluid saturation occurs (star), the pressure-temperature-composition of the silicate melt requires that the exsolved fluid phase unmixes into a low density, vapour phase and a Cl-rich brine or both phases exsolve directly from the melt simultaneously. Consequently, Cl partitions (dashed yellow line) between fluid phases and silicate melt to respect the Gibb’s phase law. Thus, the Cl concentrations in the silicate melt, vapour and brine are fixed at a buffered concentration corresponding to the thermodynamic equilibrium of the system (solid violet line); (**B**) Example data from melt from the Pollena eruption of MSV: melt inclusions (green diamonds) and residual glass (pink triangles) are reported and interpreted respect to the model as in 2a.

The equilibrium between silicate melt and fluids is generally inherited from conditions established in the reservoir rather than during magma ascent^18^. The rate of magma ascent during an eruption controls the degree to which equilibrium conditions are attained between silicate melt, vapour phase and brine at a given P. In the case of a sufficient fast ascent rate, a silicate melt may have insufficient time to re-equilibrate before fragmentation, thus preserving a record of the pre-eruptive conditions. Therefore, the Cl-buffering effect can be assessed through the analysis of residual matrix glass, representing the melt quenched at fragmentation (Fig. 2). Due to the lower diffusivity of Cl with respect to that of H_2_O in melts^36^, syn-eruptive, degassing-induced crystallisation of microlites during magma ascent^37^ could increase the Cl concentration in the residual melt. Therefore, the Cl concentration measured in the residual glass has to be corrected for the microlite effect in order to determine the pre-eruptive Cl buffer value. An alternative process which could induce Cl buffering is the saturation in a Cl-bearing crystalline phase (e.g. feldspathoids of the cancrinite-sodalite group, commonly present in the K-alkaline magmas of MSV). If Cl-bearing minerals do not represent a significant proportion of the crystallizing mineral assemblage, it is unlikely that they can induce a buffering effect on Cl concentration in the silicate melt at non-equilibrium conditions.

**Figure 3:**
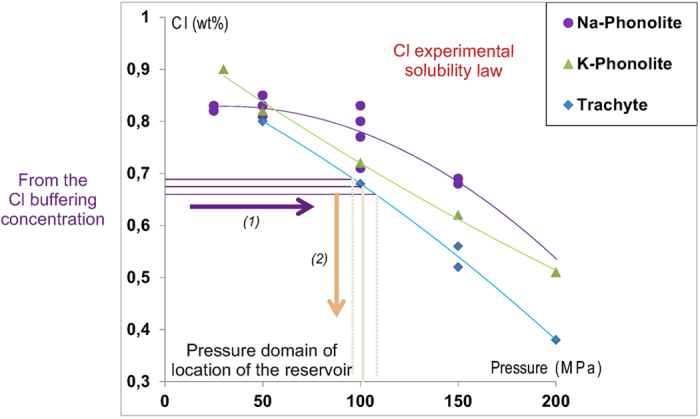
Location of the pressure domain of the magma reservoir estimated from the Cl buffer value. Experimental Cl solubility law in Na-Phonolitic^11^, K-Phonolitic^11^ and Trachytic^12^ melt saturated with aqueous fluid + brine are represented versus pressure. From the Cl buffering concentration in silicate melt, by using the relevant experimental Cl solubility law, it’s possible to estimate the pressure domain or domains at which the magma is stored at depth. The uncertainty of the pressure estimates depends on the Cl buffering concentration (mean values obtained on several pumice clasts by EPMA) and on the experimental Cl solubility law. Arrow (1): from the Cl buffer value until the experimental Cl solubility law; Arrow (2): from the experimental Cl solubility law to the pressure. Solid line: the corresponding conversion of the Cl buffer value. Dashed lines: the uncertainty from the Cl concentration determined by EPMA, that constrains the precision on the pressure domain estimate.

Using the Cl solubility law for either a given silicate melt composition, or the law for the most similar melt composition available, the pressure conditions of magma storage can be deduced by means of the determined Cl buffer value (Fig. 3). Unfortunately, such experimental data are not available for all alkaline melt compositions. As Cl solubility largely depends upon melt composition, the pressure of the magma reservoir estimated by using a Cl experimental solubility law determined on a silicate melt of different composition (in terms of alkali ratio, or Ca, Mg, Fe content) yields an additional and significant uncertainty. In order to avoid such uncertainties, it is preferable to use a more general Cl solubility model which takes into account the whole silicate melt composition, such as the one developed by Webster and collaborators^13^,^17^.

Melt inclusions (MIs) can also provide interesting information as they represent droplets of silicate melt trapped at different stages during the differentiation process^38^. MIs trapped in phenocrysts highlight Cl incompatible behaviour during fractional crystallisation until the point of fluid saturation (Fig. 2). Those trapped after fluid saturation, in the subcritical domain in which vapour and brine are in equilibrium with the silicate melt, record the pre-eruptive achievement of Cl saturation in the magma reservoir (Fig. 2).

## Results

We analysed the products of 12 explosive eruptions of MSV^37^,^39^,^40^ (Fig. 5), including four Plinian (Pomici di Base, Mercato, Avellino, Pompeii), five sub-Plinian (Greenish, AP1, AP2, Pollena, 1631 AD) and three violent Strombolian events of the recent series (1822, 1906, 1944)^39^,^41^. All magmas emitted during the MSV activity were alkali-rich melts that had undergone complex processes of differentiation, including mixing, assimilation, and fluid exsolution^40^,^42^. The residual matrix glass compositions range from tephritic to phonolitic and trachytic (Fig. 5). Many of these deposits show a variable compositional zoning, with a general decrease in melt evolution from the earliest to the latest erupted products. Such compositional zoning has been interpreted as the result of progressive tapping of a compositionally zoned reservoir^40^,^42^,^43^, accompanied by complex syn-eruptive magma mixing processes^42^. We have focused our research on the earliest erupted, most-evolved products of each event, likely representing the uppermost, fluid-saturated portion of the reservoir. Both residual matrix glass (Table S1, S2) and MI (Table S3) compositions were studied. MIs register a more complex history by exhibiting a wide range of Cl content, with values from within fluid-undersaturated conditions to within fluid-saturated conditions similar to those measured in the residual glass (Fig. 2). Here, we focus only on the residual matrix glass composition, as our interest is in the shallow plumbing system. In such glasses of the products of all eruptions, the F concentration positively correlates with the degree of melt differentiation, thus suggesting its overall behaviour as an incompatible element with minimal partitioning into a fluid. We note that CaO may also be used as a differentiation index in alkali magmas. On the contrary, the Cl content is constant, regardless of the evolving melt composition (Fig. 6; Tables S1, S2). As Cl-rich minerals are rare in the investigated rocks, the buffered Cl values, corrected for microlite crystallization, must be a result of the silicate melt being in equilibrium with a two-phase fluid. This is because the presence of a two phase fluids is the only other phase assemblage that can fix the Cl content in the system. Microlite crystallization induced by syn-eruptive degassing clearly occurred and varied from 0 to 30 vol% (Table S1), depending on the eruption.

The trachytic melt involved during the earliest-erupted Pomici di Base (22,030 yr BP) and Greenish (19,065 yr BP) eruptions, show buffered Cl contents of 6,764 ± 180 and 5,534 ± 290 ppm, respectively (Fig. 6a). The phonolitic magma of the Mercato (8,890 yr BP) eruption displays a value of 6,306 ± 320 ppm, slightly higher than those of the 5,200 ± 400 and 5,368 ± 380 ppm displayed by the other large Plinian eruptions of Avellino (4,365 yr BP) and Pompeii (AD 79), respectively (Fig. 6b). The phonolitic tephrite to phonolite melts feeding the two main AP eruptions at 3,500 yr BP (AP1 and AP2^37^), occurring shortly after the major Plinian Avellino eruption, share close Cl values of 5,475 ± 120 and 5,196 ± 490 ppm, respectively (Fig. 6b). The phonolitic tephrite melt from the Pollena (AD 472) and 1631 eruptions, the two latest sub-Plinian events, display Cl values restricted to 6,907 ± 270 and 6,688 ± 280 ppm, respectively (Fig. 6b). The tephritic melt of the more recent and violent 1822, 1906 and 1944 Strombolian eruptions also highlight the same Cl buffering effect, at values of 7,524 ± 230, 6,226 ± 190, and 5,022 ± 218 ppm, respectively (Fig. 6c).

## Discussion

The Cl solubility in silicate melt^5^, mainly dependent upon composition and P (negligible T effect), is a key parameter to convert the Cl buffer value detected in residual matrix glass into a P value indicative of the depth of the shallow magma reservoir feeding the eruption. The Cl solubility in a given melt composition can be estimated through two different methods. One method is based on the use of the experimentally determined solubility law defined on the same magma composition^11^,^12^, hereafter referred to as “experimental Cl solubility”, while the other on the use of modelling^13^,^17^, hereafter referred to as “modelled Cl solubility”. In the following we will discuss and compare the results for the studied MSV eruptions by using both methods, which will demonstrate the very strong dependence of Cl solubility on melt composition and will reveal the pre-eruptive pressure conditions at which Cl buffer was reached for the different cases analysed.

As the pre-eruptive Cl content in the different MSV melts varies in a bracket of ± 200–300 ppm, equilibrium pre-eruptive pressures estimated with the experimental Cl solubility method for each eruption, yield a small uncertainty (around 10 MPa; Fig. 3). Among the different MSV erupted magmas, Cl solubility has been experimentally measured only for the trachytic Pomici di Base^12^ and the K-phonolitic Pompeii^11^, melts. For these eruptions, the pressure domain estimated from the measured Cl buffering value in the natural samples is highly precise, and gives values of 100 ± 10 and 185 ± 10 MPa, respectively. Cl solubility curves are not available for the compositions of the magmas feeding the other studied eruptions. Using the results of experiments on the Na-phonolitic rocks of Montaña Blanca (Canary Islands)^12^, that are compositionally similar to the residual glasses of the Mercato, Avellino, AP 1, and AP 2 products, we have defined pressure domains for the magma reservoirs feeding these four eruptions of 170 ± 10, 200 ± 10, 195 ± 10 and 200 ± 10 MPa, respectively. For the Greenish eruption glass, that straddles the phonolite and trachyte fields (Fig. 5), we have estimated a large pressure domain of 150–200 MPa on the basis of the Cl solubility laws available for both trachytic and K-phonolitic melts. Cl solubility for the Pompeii K-phonolite melt has also been used for the mildly evolved glasses of both Pollena and 1631 eruptions, estimating equilibrium pressures of 105 ± 10 and 115 ± 15 MPa, respectively. For the tephritic glasses of the 1822, 1906 and 1944 eruptions, the closest available Cl solubility law, although poorly constrained, derives from experiments performed on a basaltic composition^44^. The magmas of these three eruptions show different Cl buffering values, but the same equilibrium pressure value of ~105 MPa, as imposed by the very few experimental data.

A modelled Cl solubility method, such as the one developed by Webster and collaborators^16^,^17^ (Fig. 7) takes into account composition (in particular including S content), P, and T of the investigated melt. In our study we have used the composition of each MI and the mean of the compositions of the residual matrix glass detected in variable fragments (Tables S2, S3; Fig. 7). No systematic S measurements have been performed, but literature data provide considerable constraints on S concentrations in melt. The residual matrix glasses of the large plinian eruptions (Mercato, Avellino, Pompeii) exhibit S concentrations between 200–300 ppm^45^, and those of the subplinian eruptions of Pollena^46^ and 1631^45^ between 1800–2000 and 1000–1200 ppm, respectively (EPMA data). Poor constraints exist for the Pomice di Base and Greenish eruptions that give S estimates of 500–1300 and 200–2800 ppm, respectively (EDS data^39^). For the last eruptions of 1822, 1906 and 1944, that involve less-differentiated melts, melt inclusions in olivines provide S contents of 1800–1900 ppm for 1906 and 1000–2400 ppm for 1944^47^. In addition, the K-tephritic to K-basanite magma that is thought to supply the superficial reservoir has S contents between 1500–3000 ppm^42^,^47^. The Cl saturation pressure values for the Pomici di Base and Pompeii melts, estimated by using the modelled Cl solubility taking into account S effect and the experimental Cl solubility methods (Fig. 7), are in very good agreement (Figs 3 and 6a). For the Mercato, Avellino, AP 1 and AP 2 melts, the pressure values estimated using the Cl solubility law for Na-phonolite and those derived from Cl solubility modelling taking into account S effect are in the same range. For the magmas feeding the Greenish, Pollena, 1631, 1822, 1906, and 1944 eruptions, for which experimental solubility data are not available, Cl solubility modelling (corrected for the S effect) provides better constrained pressure estimates. For the Greenish feeding reservoir, a pressure of 100 MPa gives the best fit (Fig. 7). The latest two sub-Plinian eruptions of Pollena and 1631 also highlight an equilibrium pressure for their magma reservoirs of ~100 MPa. Cl modelling requires lower equilibrium pressures for the magmas extruded during the latest three studied eruptions, of ~80 MPa for those occurred in 1822 and 1906, and ~50 MPa for the 1944 event.

Whatever the method used to estimate the Cl solubility, experimental or modelled, the pressure values we have obtained by using the buffered Cl contents of melts as a geobarometer, are in very good agreement with published data using other pressure constraints for the same MSV eruptions. The pressure domain estimated from the Cl buffering effect for the reservoirs feeding the Mercato, Avellino and Pompeii Plinian eruptions is similar to that arising from experimental petrology (200 ± 20 MPa)^48^. The value of ~100 MPa that we have estimated for the magma feeding the Pollena eruption is in agreement with the results of both experimental petrology (100 ± 20 MPa)^48^ and MI studies (~95 MPa)^45^,^46^. Also, our pressure estimates for the reservoirs feeding the post-Pompeii eruptions are in good agreement with the results of both experimental petrology and MI studies^45^,^46^,^47^,^48^. The pressure value of the magmatic reservoir of the Pomici di Base eruption deduced from the Cl-buffering effect (100 ± 10 MPa) strongly contrasts with that obtained on feldspar geobarometers (300–400 MPa)^49^ while it is coherent with the estimates resulting from water fugacity-based studies (below 165 MPa)^50^. Modelling Cl concentrations in the silicate melt at 300–400 MPa gives unrealistic values. The similar pressure value obtained for the reservoirs feeding the Pomici di Base and the Greenish eruptions^37^,^39^ (Table S1), that are very close in magma composition and dynamics, indirectly confirms the suitability of our estimate. Neither direct experimental petrology nor field (e.g. nature of accidental lithic fragments) data that could contribute to assessments of the depth of the reservoirs feeding these two eruptions are available.

The volatile solubility data presented here depict a time and space evolution of the shallow MSV plumbing system (Fig. 8) that is slightly different for that previously discussed by other authors^37^,^39^,^45^,^48^. This evolution summarizes the discussion on the choice of the best method to estimate the pressure of the shallow reservoir to establish this time and space evolution (see above and Table S4). In fact, basing on the new P estimated, the magmas emitted during the earliest high-magnitude eruptions (Pomici di Base and Greenish) were stored in a shallow reservoir located at a depth corresponding to a pressure of ~100 MPa, in contrast with what previously suggested^49^. A deepening of the magma storage to pressure conditions between ~180 and 200 MPa began with the formation of the reservoir of the Mercato eruption (between 19,265 and 8,890 yrs BP) and lasted at least until the Pompeii eruption (79 AD). From the Pollena (472 AD) until the 1631 eruption, the magma storage depth switched again to a shallower level (corresponding to a pressure of ~100 MPa), similar to that of the Pomici di Base event. Subsequently, it progressively raised towards more and more shallow depth, up to the one corresponding to ~50 MPa, that fed the less intense, violent Strombolian eruptions of 1822, 1906 and 1944. The post-9 ka evolution of the magmatic system confirms the previous models for this period of activity^48^. On the basis of our results, we suggest that the MSV volcanic system has been characterised through time by two main zones of magma storage, with the roof of the reservoirs located at ~180–200 (~7–8 km depth) and ~100 MPa (~3–4 km depth). The lower pressure values are consistent with petrologic constraints^38^,^51^,^52^. The higher pressure values, in good agreement with experimental petrology data^48^, are also consistent with the results of seismic surveys, suggesting the presence of a still active large reservoir with the top at ~8 km depth^53^. Finally, recent electrical resistivity investigations suggest the presence of residual cooling magma batches at 3–4 km depth, in addition to the deeper one^54^.

**Figure 4:**
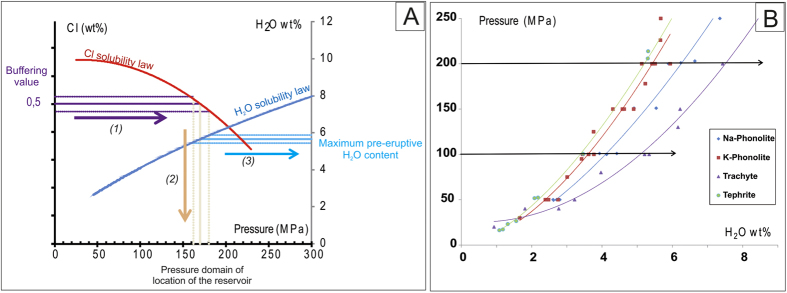
Pre-eruptive H_2_O content estimates from Cl buffer value. How to convert a Cl buffer value into a pre-eruptive dissolved H_2_O content of a melt? (**A**) From the domain of pressure deduced from the Cl buffer value, the H_2_O concentration in the silicate melt is deduced by reading the H_2_O content at this pressure using the relevant experimental H_2_O solubility law. Solid line: the corresponding conversion of the mean Cl or H_2_O value. Dashed lines: the uncertainty for the estimated Cl or H_2_O concentration. Theoretical Cl (blue) and H_2_O (red) experimental solubility laws are reported. (**B**) Experimental H_2_O solubility law for Na-phonolite^29^, K-phonolite^28^, trachyte^30^ and tephrite^31^ are reported. As for Cl, the key point is to have the H_2_O solubility laws that correspond to the studied melt composition; this constrains the uncertainty of the method as the Cl content is accurately measured by EPMA and the analytical contribution to uncertainty is relatively minor.

**Figure 5:**
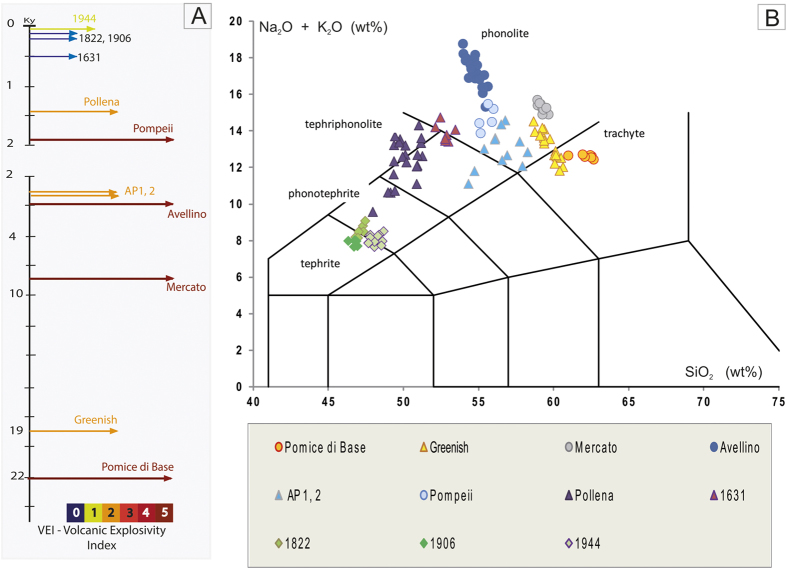
The main explosive eruptions of Mount Somma-Vesuvius (**A**) Schematic chronogram of Mount Somma-Vesuvius activity for studied explosive eruptions (modified from reference^37^). Length and colour of arrows refer to the estimated VEI. Breaks in the chronogram mark changes of timescale. (**B**) Residual glass compositions are reported in a total alkalis-silica diagram. These data refer to mean glass compositions of samples (at least 10 analyses per pumice), from the density mode of each eruptive unit described for each eruption, in which the Cl buffering effect has been identified (Table S1); except for Pompeii where the mean composition for each eruptive unit is represented.

**Figure 6:**
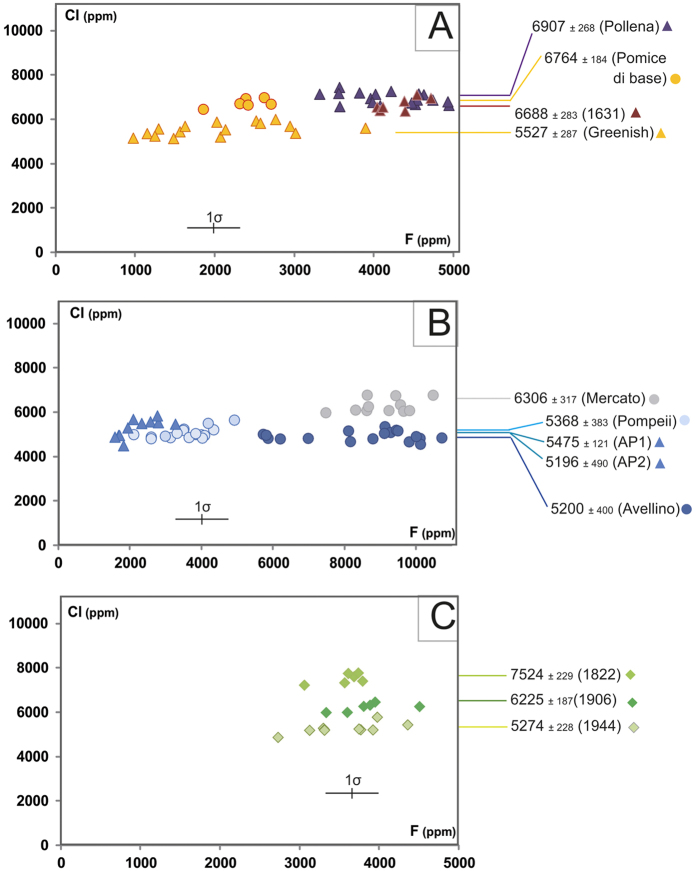
Cl vs. F contents in residual glass from the first eruptive units of each explosive eruption of Mount Somma–Vesuvius in which the Cl buffer effect has been identified. One point represents a mean Cl content of one single fragment describing the density mode (at least 10 analyses); except for Pompeii (melt inclusions + mean Cl content for each eruptive unit). Data for Pompeii^58^ and Avellino^59^ are also reported. Melt inclusion data are not represented except for Pompeii (Table S2; see discussion in the text). (**A**) Pomici di Base, Greenish, Pollena and 1631; (**B**) Mercato, Avellino, AP1, 2 and Pompeii; (**C**) 1822, 1906, 1944. Cl uncertainty is within symbol size, contrary to F (see symbol).

**Figure 7:**
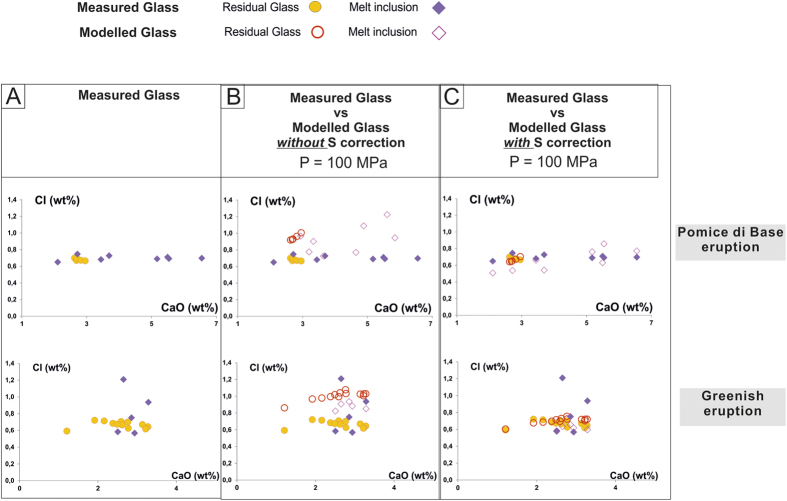
Modelled and measured Cl concentrations vs. CaO in glass (residual glass and melt inclusions) from two eruptions of MSV, Pomice di Base and Greenish eruptions. (**A**) Measured Cl concentration in residual glass (mean values of at least 10 analyses) and melt inclusions (single measurement). (**B**) Measured Cl concentration compared to modelled Cl content without S correction, at a pressure of 100 MPa. (**C**) Measured Cl concentration compared to modelled Cl content with S correction (reduction of 30% in relative of Cl solubility^17^), at the same pressure of B (100 MPa).

**Figure 8:**
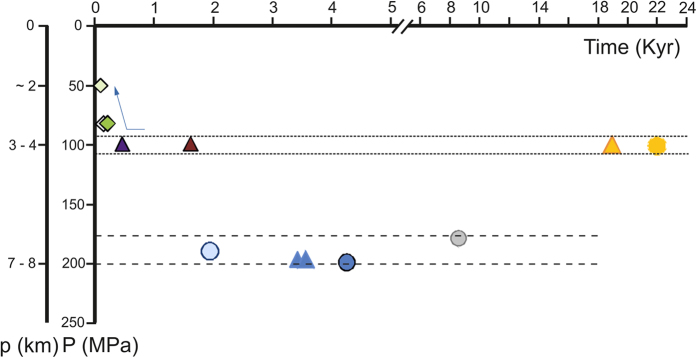
The evolution through time of the shallow plumbing system for the last 22 ky period of activity of Mount Somma–Vesuvius. The uncertainty in the pressure determination is within symbol size (see text for explanation; Table S1 and S4).

As a corollary to these new and robust constraints on the depth of the shallow plumbing system, determined by the recognition of the Cl buffering effect on the MSV products, a first estimate of the maximum pre-eruptive H_2_O content in all these silicate melts can also be derived (Fig. 4; Table S1). We estimate H_2_O concentrations using a H_2_O solubility law in the corresponding melt at the pressure deduced from the Cl-buffering effect. These first estimates give between 4.8 and 7 wt% H_2_O for the phonolitic and trachytic magmas, and 3.5 and 4.8 wt% for the less differentiated tephritic and phonotephritic melts. This approach requires that the H_2_O solubility law for the composition of the erupted magma is known, and it neglects the influences of other volatiles on H_2_O solubility (mainly CO_2_). Since CO_2_ is not considered into the H_2_O solubility law, and as CO_2_ may influence H_2_O solubility^35^, these estimates are first estimate (maximum values), as in particular the presence of CO_2_ could possibly depress the amount of water dissolved in glass at saturation, by dilution of the fluid at low bulk Cl concentration^35^, introducing consequently large error on H_2_O estimates. However, volatile studies on the products of the AD 79 Pompeii eruption confirm such values, also revealing the absence of an important CO_2_ component^55^. It is also likely that CO_2_ fluxing through the shallow magma chambers represents a significant process at MSV, as suggested by oxygen isotope data on olivine and diopsidic clinopyroxene^56^. This process could be responsible for an early establishment of volatile saturation in the magma, due to the decrease in H_2_O solubility induced by the changes in fugacity of different volatile species in the fluid phase as CO_2_ increases. This effect could be relevant especially in small magma chambers, such as those feeding the post-Pompeii mafic activity^31^,^45^. Furthermore, it could also explain the achievement of Cl buffering for such mafic compositions, as H_2_O would be forced to exsolve in response to CO_2_ fluxing^16^,^56^. Compared to H_2_O contents measured directly in melt inclusions, the minimum exsolved H_2_O content during magma ascent may be estimated. In several cases, the H_2_O contents measured in melt inclusions are consistent with our estimates (Table S1).

## Conclusion

A reliable assessment of volcanic hazard requires the time, place, and scenario of the next eruption to be defined. Facing this issue, the Cl content of evolved, Cl-enriched alkaline melts represented by matrix glasses may be used as (1) a geobarometer for locating a crustal reservoir and (2) an indicator of H_2_O dissolved content in silicate melt prior to eruption. The Cl geobarometer here presented may be useful for a large composition domain of alkaline magmas, such as rhyolite, although this was not included in our study^15^. When systematically applied to the products of the past activity of a given volcano, this geobarometer can also constrain the temporal evolution of its plumbing system. All these parameters are of interest for alkali magmas as they may allow a better understanding of storage conditions (pressure, depth and pre-eruptive volatile content) that are linked to eruptive style as highlighted for phonolitic-trachytic magmas^57^. Our results also highlight that fluid phase relations are key parameters to explain the H_2_O-CO_2_-S-Cl dissolution in a silicate melt at relevant magmatic pressures. Unfortunately, not all these relationships, such as the influence of CO_2_, S or F, are yet clearly defined. This also has implications for acquiring a deeper understanding of halogen fractionation in fluid-saturated magmas.

## Methodology

For the products of each investigated eruption, we selected one representative outcrop including the basal fallout sequence of the entire pyroclastic succession and collected samples from each major eruptive unit. Samples from the deposits of pyroclastic flow phases were not included in our study. The sampling strategy was based on the collection of at least 100 pumice clasts per eruptive unit. Each clast was then sawed into three parts, and a density measurement performed on one third of each of them by the “three weights” method^58^,^59^. Several pumice clasts representative of the eruptive unit were chosen in the mode of the density distribution, that is among those with the most representative density value (generally ~80% of the total erupted magma). Electron microprobe (EPMA) analyses (Cameca SX 100, Camparis - France) were performed on one third of the selected pumice^58^,^59^, with an acceleration voltage of 15 kV and a beam current of 4 nA. The dwell time was 10 s, except for Si (5 s) and Na (5 s). F and Cl were measured with a dwell time of 60 s, an acceleration voltage of 15 kV and a beam current of 40 nA. Analytical conditions (in particular dwell times) were established to minimize Na and F diffusion during EPMA analyses using glass standards. For inter-calibration of EPMA sessions, three natural obsidians were analysed^58^,^59^. Microlite contents of residual glass may be estimated by chemical mapping^59^. MI studies were performed on phenocrysts separated from several clasts per each studied eruptive unit. In order to have a consistent dataset, only MIs hosted in phenocrysts with diameter >125 μm, were chosen.

The Cl modelling was based on the model published by Webster and collaborators^13^ expressing the influence of major elements on Cl solubility through the definition of association coefficients linking Cl to cations in melt. Their Excel™ spreadsheet allows one to introduce and account for each matrix glass and MI composition determined by EPMA measurements (Si, Ti, Al, Fe (as FeO), Mg, Mn, Ca, Na, K, F) at a given temperature-pressure condition. The result of the computation is the modelled Cl concentration in the silicate melt. The S effect is introduced manually by reducing the Cl contents of the melts by 30 relative percent, after calculating the Cl solubility for the correct bulk melt composition, P, and T. This option is not routine in the version of the Excel™ spreadsheet because it would need more systematically run experiments with S for other melts than phonolitic in composition. This estimate of S effect is based on experiments performed by Webster and collaborators^16^,^17^ on phonolitic melt with S content (2000 ppm) in the range of that of MSV magmas.

## Additional Information

**How to cite this article**: Balcone-Boissard, H. *et al*. Chlorine as a geobarometer for alkaline magmas: Evidence from a systematic study of the eruptions of Mount Somma - Vesuvius. *Sci. Rep.* **6**, 21726; doi: 10.1038/srep21726 (2016).

## Supplementary Material

Supplementary Information
