# Supplementary material for: Chlorine as a geobarometer for alkaline magmas: Evidence from a systematic study of the eruptions of Mount Somma-Vesuvius
Source: Sci Rep. 2016 Feb 18;6:21726. doi: 10.1038/srep21726 (PMC4757863; doi:10.1038/srep21726)
Supplement: Supplementary Information [file srep21726-s1.pdf]

Chlorine as a geobarometer for alkaline magmas: Evidence from a systematic study of the eruptions of Mount Somma-Vesuvius

H. Balcone-Boissard[1*], G. Boudon[2], R. Cioni[3], J.D. Webster[4], G. Zdanowicz[2,5], G. Orsi[5,6], L. Civetta[5,7]

* Correspondence to helene.balcone_boissard@upmc.fr

Supplementary information

Table S1: Synthesis of data obtained on the successive eruptions studied

Name	Age	Melt composition	Cl buffer	Microlite	Cl solubility law	Pressure	Pressure	H_2O	Litterature H_2O data
				%		MPa	MPa	wt%	wt%
						Experim.	*Modelling*	*Cl buffer*	
PdB	22,030±175 yr cal BP	trachyte	6764 ±184	0	Pomice di base melt [14]	100 ±10	100	4,8	1,4 - 3,8 [48]
Greenish	19,065±105 yr cal BP	trachyte - phonolite	5534 ±290	20	Between trachyte [14] and Na phonolite [13]	150 - 200	100	5,2 - 7	4 - 5 [44]
Mercato	8,890±90 yr cal BP	Na phonolite	6306 ±317	0	Na-phonolite Montana blanca [13]	175 ±10	180	5,7	7 [57]
Avellino	4,365±40 yr cal BP	Na phonolite	5200 ±400	0	Na-phonolite Montana blanca [13]	200 ±10	200	6,2	2,3 - 5,3 [34]
AP1	3,500±60 yr cal BP	Na phonolite	5474 ±121	0	Na-phonolite Montana blanca [13]	195 ±10	195	6,1	no
AP2	3,500±40 yr cal BP	Na phonolite	5195 ±490	0	Na-phonolite Montana blanca [13]	200 ±10	200	6,2	no
Pompeii	AD 79	K phonolite	5368 ±383	30	K phonolite Pompeii [13]	185 ±10	185	5,1	1 - 6,5 [37]
Pollena	AD 472	tephriphonolite	6907 ±268	20	K phonolite [13]	105 ±10	100	3,7 ± 0,2	2 - 3,6 [44]
1631	1631	tephriphonolite	6687 ±283	20	K phonolite [13]	115 ±15	100	3,9 ± 0,3	3 - 3,3 [46]
1822	1822	tephrite - phonotephrite	7524 ±229	0	Tephrite [44]	107 ± 0	80	3,5	3,3-4,4 [45]
1906	1906	tephrite - phonotephrite	6226 ±187	0	Tephrite [44]	107 ± 0	80	3,5	3-3,2 [45]
1944	1944	tephrite - phonotephrite	5273 ±228	0	Tephrite [44]	107 ± 0	50	3,5	2,3 -3,4 [45]

1. Eruption; 2. Age; 3. Melt composition (residual glass); 4. Cl buffering value (ppm), corrected from microlite content; 5. Microlite content; 6. Experimental Cl solubility law; 7. Pressure domain (MPa) deduced from Cl (this study); 8. Experimental H_2O solubility law; 9.Pre-eruptive H_2O content deduced from Cl (this study); 10. Pre-eruptive H_2O content from literature.

Table S2

Pomice di Base

White pumice phase

Eruptive unit	PdB0		PdB1		PdB1		PdB2		PdB2		PdB 4a	
Sample	3		1		11		2		7		69	
n	11	σ (%)	9	σ (%)	9	σ (%)	11	σ (%)	11	σ (%)	3	σ (%)
SiO2	62,53	0,38	62,43	0,24	62,65	0,29	62,49	0,28	62,06	0,35	61,92	0,48
TiO2	0,37	0,07	0,33	0,06	0,29	0,07	0,30	0,12	0,36	0,08	0,31	0,08
Al2O3	18,31	0,15	18,43	0,15	18,35	0,20	18,28	0,16	18,35	0,32	18,00	0,28
Fe2O3	3,04	0,14	3,04	0,23	3,11	0,21	3,22	0,13	3,31	0,20	3,50	0,21
MnO	0,14	0,04	0,19	0,04	0,18	0,06	0,16	0,06	0,18	0,07	0,19	0,04
MgO	0,25	0,03	0,23	0,04	0,25	0,04	0,25	0,03	0,27	0,03	0,33	0,01
CaO	2,68	0,11	2,63	0,09	2,68	0,08	2,70	0,07	2,82	0,09	2,96	0,05
Na2O	4,51	0,21	4,58	0,26	4,33	0,22	4,33	0,19	4,47	0,22	4,46	0,12
K2O	8,13	0,20	8,12	0,22	8,12	0,34	8,24	0,23	8,13	0,25	8,27	0,16
P2O5	0,05	0,03	0,03	0,02	0,03	0,06	0,03	0,04	0,05	0,03	0,07	0,07
total	100		100		100		100		100		100	
F (ppm)	2378	635	2620	769	2707	574	2389	679	2313	558	2417	772
Cl (ppm)	6873	297	6991	246	6697	176	6947	118	6720	160	6656	183
Na2O+K2O	13	0,23	13	0,22	12	0,17	13	0,28	13	0,36	13	0,23
Na/K	1,0		1,0		0,9		0,9		1,0		0,9	
AI	0,7		0,7		0,7		0,7		0,7		0,7	

Greenish

First pumice phase

Eruptive unit	VV2		VV2		VV2		VV2		VV2		VV3	
Sample	3		7		21		32		99		1	
n		σ (%)		σ (%)		σ (%)		σ (%)		σ (%)		σ (%)
SiO2	59,34	0,43	60,48	0,24	59,36	0,40	60,61	0,66	59,36	0,38	58,74	0,45
TiO2	0,34	0,06	0,35	0,07	0,31	0,06	0,34	0,06	0,35	0,05	0,40	0,08
Al2O3	19,27	0,16	19,45	0,19	19,27	0,31	19,44	0,15	19,10	0,18	18,60	0,40
Fe2O3	4,14	0,26	4,04	0,13	4,09	0,28	3,95	0,31	4,05	0,16	5,04	0,79
MnO	0,16	0,02	0,18	0,03	0,18	0,05	0,15	0,03	0,17	0,04	0,26	0,04
MgO	0,30	0,02	0,26	0,02	0,26	0,05	0,28	0,04	0,29	0,03	0,29	0,11
CaO	2,78	0,18	2,59	0,59	2,38	0,75	3,14	0,48	3,28	0,13	2,62	0,79
Na2O	5,59	0,28	5,21	0,60	6,20	0,82	4,37	0,71	4,65	0,25	5,57	0,73
K2O	8,06	0,13	7,34	0,33	7,91	0,26	7,67	0,41	8,67	0,31	8,39	0,47
P2O5	0,03	0,05	0,09	0,03	0,04	0,03	0,05	0,03	0,07	0,03	0,07	0,04
total	100,00	0,00	100,00	0,00	100,00	0,00	100,00	0,00	100,00	0,00	100,00	0,00
F (ppm)	2589	395	2530	598	3680	833	1616	441	1437	260	2667	484
Cl (ppm)	6519	590	7360	1242	7125	687	6975	636	6712	150	6930	283
Na2O+K2O	13,65	0,37	12,55	0,66	14,11	0,76	12,05	0,44	13,33	0,33	13,96	1,05
Na/K	1,21		1,24		1,37		1,00		0,93		1,15	
AI	0,7		0,6		0,7		0,6		0,7		0,8	

Eruptive unit	VV3		VV3		VV3		VV5		VV5		VV5		VV5	
Sample	8		43		86		6		7		8		12	
n		σ (%)		σ (%)		σ (%)		σ (%)		σ (%)		σ (%)		σ (%)
SiO2	58,65	0,55	60,19	0,69	60,12	0,34	59,23	0,95	60,03	0,63	60,14	0,82	59,04	0,51
TiO2	0,37	0,03	0,31	0,08	0,34	0,06	0,35	0,07	0,34	0,07	0,31	0,07	0,40	0,09
Al2O3	18,88	0,26	19,27	0,43	19,52	0,32	18,96	0,31	19,45	0,62	19,28	0,36	19,32	0,12
Fe2O3	5,05	0,71	3,93	0,22	4,49	0,57	4,26	0,33	3,81	0,82	4,28	0,65	3,75	0,00
MnO	0,27	0,05	0,13	0,03	0,21	0,06	0,16	0,04	0,13	0,05	0,18	0,06	0,15	0,04
MgO	0,26	0,04	0,28	0,03	0,23	0,06	0,30	0,02	0,28	0,05	0,29	0,03	0,18	0,09
CaO	1,92	0,14	3,23	0,11	2,16	0,44	2,49	0,24	3,22	0,18	2,76	0,56	1,21	0,56
Na2O	6,20	0,41	4,08	0,61	5,74	0,63	5,93	1,37	4,41	0,65	6,09	1,08	8,29	0,21
K2O	8,34	0,34	8,54	0,33	7,18	0,26	8,25	0,76	8,28	0,28	6,60	0,56	7,59	0,15
P2O5	0,08	0,03	0,05	0,05	0,03	0,03	0,06	0,03	0,06	0,04	0,07	0,03	0,07	0,01
total	100,00	0,00	100,00	0,00	100,00	0,00	100,00	0,00	100,00	0,00	100,00	0,00	100,00	0,00
F (ppm)	3454	275	1218	300	3149	331	4867	996	1852	178	3216	663	2946	915
Cl (ppm)	7508	424	6460	220	7428	999	7018	683	6434	350	7296	453	6170	2422
Na2O+K2O	14,54	0,30	12,62	0,74	12,91	0,47	14,18	0,83	12,69	0,47	12,69	0,77	15,88	0,07
Na/K	1,30		0,83		1,40		1,27		0,93		1,63		1,90	
AI	0,8		0,7		0,7		0,7		0,7		0,7		0,8	

Mercato

First pumice phase

Eruptive unit	VM 2		VM 2		VM 3		VM3		VM4		VM4		VM4	
Sample	2		5		8		11		5		9		2	
n	10	σ (%)	10	σ (%)	9	σ (%)	3	σ (%)	15	σ (%)	11	σ (%)	10	σ (%)
SiO2	58,93	0,24	59,31	0,65	59,30	0,39	59,65	0,89	59,12	0,50	59,24	0,96	58,90	0,82
TiO2	0,11	0,10	0,11	0,07	0,11	0,08	0,04	0,04	0,12	0,06	0,09	0,11	0,13	0,09
Al2O3	21,53	0,27	21,61	0,35	21,48	0,33	21,36	0,63	21,35	0,27	21,30	0,37	21,32	0,26
Fe2O3	2,11	0,14	2,05	0,16	2,04	0,11	2,14	0,17	2,12	0,19	2,31	0,15	2,16	0,44
MnO	0,24	0,04	0,17	0,04	0,17	0,08	0,24	0,04	0,22	0,06	0,18	0,05	0,19	0,07
MgO	0,04	0,02	0,06	0,02	0,04	0,02	0,05	0,04	0,05	0,02	0,05	0,02	0,04	0,03
CaO	1,60	0,14	1,61	0,05	1,55	0,04	1,57	0,13	1,52	0,07	1,58	0,10	1,56	0,29
Na2O	8,62	0,45	8,45	0,92	8,56	0,21	8,47	1,55	9,22	0,51	8,98	1,06	9,44	0,57
K2O	6,80	0,36	6,61	0,18	6,72	0,20	6,45	0,26	6,26	0,22	6,24	0,21	6,28	0,28
P2O5	0,03	0,03	0,02	0,04	0,03	0,03	0,03	0,07	0,00	0,03	0,03	0,03	-0,01	0,06
total	100		100		100		100		100		100		100	
F (ppm)	10467	1424	9552	1883	9421	1281	8625	1618	9642	1373	8301	1767	9808	1412
Cl (ppm)	6781	487	6347	323	6763	763	6785	615	6059	382	6110	1007	6087	1387
Na2O+K2O	15,41	0,33	15,06	0,90	15,28	0,18	14,92	1,54	15,49	0,54	15,22	1,06	15,71	0,75
Na/K	2,21		2,22		2,22		2,29		2,56		2,51		2,62	
AI	0,7		0,7		0,7		0,7		0,7		0,7		0,7	

Eruptive unit	VM5		VM5		VM6	
Sample	5		3		2	
n	5	σ (%)	4	σ (%)	12	σ (%)
SiO2	59,24	0,58	59,31	0,64	59,04	0,48
TiO2	0,15	0,05	0,11	0,08	0,15	0,05
Al2O3	21,57	0,52	21,31	0,27	21,26	0,28
Fe2O3	2,21	0,06	2,21	0,19	2,13	0,26
MnO	0,17	0,04	0,19	0,06	0,16	0,07
MgO	0,08	0,02	0,06	0,02	0,06	0,01
CaO	1,63	0,07	1,64	0,10	1,66	0,06
Na2O	8,65	1,12	8,83	0,46	8,88	0,45
K2O	6,27	0,20	6,35	0,21	6,66	0,37
P2O5	0,04	0,02	-0,01	0,04	0,00	0,03
total	100		100		100	
F (ppm)	8613	1045	7469	1282	8660	1231
Cl (ppm)	6092	329	5990	269	6265	292
Na2O+K2O	14,91	1,14	15,18	0,40	15,53	0,49
Na/K	2,40		2,42		2,33	
AI	0,7		0,7		0,7	

Avellino data from reference 64

Eruptive unit	EU1a		EU1a		EU1a		EU1b		EU1b		EU1b		EU1b	
Sample	2		1		4		73		13		58		57	
n	13	σ (%)	5	σ (%)	9	σ (%)	14	σ (%)	12	σ (%)	19	σ (%)	16	σ (%)
SiO_2	55,27	0,78	54,33	1,18	54,76	1,07	55,23	0,83	54,13	1,23	54,76	0,78	54,70	0,45
TiO_2	0,22	0,10	0,12	0,09	0,24	0,11	0,15	0,11	0,12	0,07	0,12	0,09	0,10	0,05
Al_2O_3	21,93	0,38	21,41	0,42	21,44	0,41	23,12	0,52	22,66	0,63	22,65	0,39	22,76	0,25
Fe_2O_3	2,08	0,35	2,33	0,35	2,17	0,26	1,53	0,26	1,48	0,26	1,37	0,14	1,37	0,16
MnO	0,10	0,06	0,13	0,06	0,11	0,10	0,11	0,08	0,17	0,06	0,13	0,06	0,14	0,09
MgO	0,17	0,06	0,21	0,07	0,17	0,02	0,05	0,02	0,04	0,02	0,04	0,02	0,05	0,02
CaO	2,90	0,60	3,25	0,62	3,13	0,87	1,52	0,24	1,45	0,23	1,36	0,11	1,50	0,31
Na_2O	8,46	0,63	9,55	1,08	8,61	0,59	9,90	0,74	11,49	1,31	10,80	0,67	10,60	0,47
K_2O	7,63	0,74	7,36	0,98	8,27	0,66	6,51	0,24	6,37	0,46	7,38	0,35	7,35	0,25
P_2O_5	0,16	0,27	0,12	0,14	0,22	0,29	0,02	0,05	0,03	0,06	0,02	0,03	0,02	0,03
F (ppm)	4608	0,17	4522	0,26	2551	0,14	9155	0,12	9120	0,18	5855	0,16	6197	0,17
Cl (ppm)	4685	0,04	4375	0,06	4214	0,13	5171	0,04	5364	0,07	4837	0,08	4810	0,06
Total	99,85		99,70		99,81		99,56		99,40		99,71		99,70	
Na_2O+K_2O	16,09	0,94	16,91	1,54	16,88	0,98	16,41	0,82	17,86	1,38	18,18	0,57	17,95	0,48
Na/K	1,9	0,26	2,3	0,37	1,8	0,18	2,6	0,20	3,1	0,41	2,5	0,23	2,5	0,15
AI	0,7		0,8		0,8		0,7		0,8		0,8		0,8	

Eruptive unit	EU2a		EU2a		EU2a		EU2b	
Sample	106		109		63		4	
n	5	σ (%)	6	σ (%)	3	σ (%)	7	σ (%)
SiO_2	53,98	1,18	54,62	0,48	54,85	0,44	54,88	0,59
TiO_2	0,14	0,09	0,14	0,04	0,11	0,04	0,06	0,09
Al_2O_3	22,79	0,60	23,79	0,58	23,52	0,38	23,16	0,14
Fe_2O_3	1,43	0,14	1,30	0,22	1,45	0,15	1,41	0,10
MnO	0,13	0,02	0,18	0,05	0,13	0,03	0,10	0,06
MgO	0,03	0,01	0,02	0,01	0,02	0,00	0,04	0,01
CaO	1,32	0,38	1,27	0,05	1,14	0,05	1,32	0,19
Na_2O	11,56	1,07	10,20	0,58	10,76	0,57	11,10	0,76
K_2O	6,68	0,51	6,78	0,34	6,18	0,03	6,07	0,26
P_2O_5	0,03	0,01	0,02	0,02	0,05	0,02	ε	0,03
F (ppm)	9796	0,00	9281	0,01	9438	0,01	9104	0,16
Cl (ppm)	4679	0,12	5101	0,12	5205	0,16	5061	0,06
Total	99,53		99,74		99,68		99,56	
Na_2O+K_2O	18,23	1,34	16,98	0,69	16,94	0,56	17,17	0,69
Na/K	3,02	0,27	2,62	0,19	3,03	0,17	3,2	0,31
AI	0,8		0,7		0,7		0,7	

AP

Eruptive unit	AP1 middle		AP1 middle		AP1 middle		AP1 middle	
Sample	12		37		24		33	
n	7	σ (%)	8	σ (%)	3	σ (%)	5	σ (%)
SiO2	56,90	0,86	57,91	1,20	56,10	0,91	56,08	0,37
TiO2	0,43	0,11	0,30	0,05	0,37	0,08	0,40	0,16
Al2O3	20,29	0,77	21,02	0,72	20,59	1,17	20,09	0,37
Fe2O3	4,68	0,71	3,77	1,32	3,97	1,35	4,63	0,30
MnO	0,22	0,04	0,14	0,10	0,15	0,14	0,20	0,02
MgO	0,31	0,12	0,16	0,06	0,57	0,49	0,42	0,08
CaO	3,18	1,49	3,43	1,13	3,68	2,15	3,34	0,40
Na2O	7,34	2,25	7,04	1,04	5,31	2,14	7,12	0,22
K2O	5,34	1,21	5,04	1,50	8,31	5,49	6,42	0,36
P2O5	0,16	0,07	0,21	0,22	0,08	0,08	0,11	0,10
total	100,00	0,00	100,00	0,00	100,00	0,00	100,00	0,00
F (ppm)	5579	1405	5475	1814	5305	1044	5541	523
Cl (ppm)	2572	986	3270	1450	1939	714	2794	1035
Na2O+K2O	12,68	1,78	12,08	1,49	13,62	3,37	13,55	0,51
Na/K	2,59		2,62		1,59		1,93	
AI	0,6		0,6		0,7		0,7	

Eruptive unit	AP2 base		AP2 base		AP2 base		AP2 base	
Sample	0		27		10		2	
n	5	σ (%)	11	σ (%)	13	σ (%)	13	σ (%)
SiO2	58,3	0,3	57,7	0,4	56,8	0,4	56,5	0,7
TiO2	0,3	0,1	0,3	0,1	0,4	0,1	0,4	0,1
Al2O3	20,9	0,2	20,7	0,2	20,5	0,4	20,4	0,3
Fe2O3	3,4	0,4	3,5	0,1	3,6	0,3	3,8	0,3
MnO	0,0	0,1	0,1	0,1	0,1	0,1	0,1	0,1
MgO	0,3	0,1	0,3	0,0	0,3	0,0	0,4	0,0
CaO	3,0	0,2	2,8	0,2	2,8	0,2	3,1	0,2
Na2O	4,9	0,5	5,2	0,4	5,3	0,3	5,0	0,6
K2O	7,9	0,1	8,2	0,2	9,3	0,2	9,3	0,4
P2O5	0,1	0,1	0,1	0,1	0,1	0,1	0,1	0,1
total	100,0	0,0	100,0	0,0	100,0	0,0	100,0	0,0
F (ppm)	4972	528	4963	215	4503	383	4889	377
Cl (ppm)	1685	222	1706	425	1820	223	1580	240
Na2O+K2O	12,9	0,5	13,4	0,5	14,6	0,3	14,4	0,6
Na/K	1,1		1,1		1,0		0,9	
AI	0,6		0,6		0,7		0,7	

Pompeii data from reference 63; mean value represented

White pumice phase

Eruptive unit	U1		U2		U3		U4	
Sample								
n	4	σ (%)	22	σ (%)	10	σ (%)	6	σ (%)
SiO2	55,04	1,21	55,89	0,87	55,36	0,79	56,22	1,12
TiO2	0,25	0,13	0,21	0,09	0,22	0,08	0,24	0,10
Al2O3	23,42	0,39	23,04	0,46	23,22	0,58	22,70	0,57
Fe2O3	2,88	0,12	2,36	0,25	2,25	0,21	2,62	0,34
MnO	0,28	0,14	0,15	0,08	0,16	0,06	0,08	0,06
MgO	0,12	0,03	0,10	0,03	0,10	0,03	0,17	0,04
CaO	3,16	0,14	2,95	0,19	3,11	0,16	3,49	0,22
Na2O	8,85	1,58	7,82	0,66	8,65	0,42	7,73	1,42
K2O	5,99	0,32	7,44	0,33	6,88	0,42	6,70	0,23
P2O5	nd	nd	0,08	0,04	0,16	0,06	0,08	0,04
total	100		100		100		100	
F (ppm)	4688	1610	4030	1001	4660	895	2691	864
Cl (ppm)	7365	1010	6433	521	6172	1160	5715	1010
Na2O+K2O	14,84		15,27		15,53		14,43	
Na/K	2,57		1,83		2,19		2,01	
AI	0,6		0,7		0,7		0,6	

Pollena

First pumice phase

Eruptive unit	VP3		VP3		VP5		VP5		VP5		VP5	
Sample	8		23		34		20		17		1	
n	11	σ (%)	12	σ (%)	7	σ (%)	6	σ (%)	9	σ (%)	12	σ (%)
SiO2	50,86	1,06	51,74	0,33	50,18	1,44	50,58	1,24	51,36	0,43	50,77	0,74
TiO2	0,45	0,12	0,47	0,10	0,45	0,15	0,47	0,17	0,48	0,07	0,49	0,11
Al2O3	21,95	0,63	22,03	0,32	21,32	0,78	21,97	0,81	22,33	0,28	21,47	0,37
Fe2O3	6,12	0,75	6,15	0,19	6,59	1,26	6,25	1,32	6,03	0,59	6,57	0,55
MnO	0,23	0,04	0,24	0,04	0,20	0,11	0,19	0,08	0,21	0,04	0,27	0,03
MgO	0,61	0,30	0,43	0,09	0,82	0,39	0,64	0,28	0,67	0,09	0,70	0,19
CaO	6,77	1,42	6,09	0,40	6,78	1,42	5,99	1,40	6,61	0,43	6,79	0,66
Na2O	8,37	1,11	8,63	0,44	7,98	1,00	8,30	1,00	8,04	0,84	8,44	0,43
K2O	4,53	0,85	4,13	0,44	5,48	0,86	5,49	0,83	4,19	0,48	4,37	0,32
P2O5	0,11	0,11	0,08	0,04	0,19	0,17	0,13	0,13	0,08	0,09	0,13	0,06
total	100		100		100		100		100		100	
F (ppm)	6379	1152	5650	347	6432	1089	6177	1983	4147	1046	6161	621
Cl (ppm)	8692	572	8357	632	8115	637	8301	951	8933	855	8504	567
Na2O+K2O	12,71	1,45	12,64	0,23	13,25	1,15	13,56	1,48	12,13	0,83	12,60	0,61
Na/K	3,31		3,69		2,59		2,65	0,60	3,35	0,41	3,43	0,26
AI	0,6		0,6		0,6		0,6		0,5		0,6	

Eruptive unit	VP6		VP6		VP6		VP6	
Sample	14		58		2		87	
n	13	σ (%)	12	σ (%)	9	σ (%)	3	σ (%)
SiO2	50,95	0,88	50,54	0,79	51,35	0,28	50,14	3,14
TiO2	0,46	0,08	0,51	0,10	0,49	0,05	0,53	0,18
Al2O3	21,91	0,41	21,75	0,46	22,41	0,33	21,52	1,11
Fe2O3	6,11	0,63	6,24	0,57	6,09	0,48	6,97	2,08
MnO	0,20	0,05	0,24	0,07	0,24	0,05	0,20	0,07
MgO	0,64	0,17	0,74	0,14	0,61	0,16	0,85	0,58
CaO	6,17	0,82	6,25	0,45	6,50	0,74	7,79	2,89
Na2O	8,27	0,54	8,21	0,36	8,54	0,69	7,30	0,71
K2O	5,17	0,57	5,40	0,37	3,65	0,38	4,54	0,74
P2O5	0,13	0,12	0,12	0,08	0,11	0,06	0,16	0,14
total	100		100		100		100	
F (ppm)	5924	813	5783	714	4946	649	5679	700
Cl (ppm)	8600	756	8900	832	8688	671	8606	513
Na2O+K2O	13,22	0,89	13,39	0,54	12,07	0,97	11,64	1,39
Na/K	2,81	0,29	2,66	0,21	4,10	0,36	2,82	0,29
AI	0,6		0,6		0,5		0,6	

1631

First pumice phase

Eruptive unit	B		B		B		C		C		C		D	
Sample	17		22		21		29		13		17		12	
n	10	σ (%)	9	σ (%)	12	σ (%)	20	σ (%)	10	σ (%)	10	σ (%)	10	σ (%)
SiO2	53,78	1,08	53,68	0,44	53,61	1,00	53,32	0,48	54,33	0,43	53,57	0,57	52,95	1,10
TiO2	0	0,14	0,67	0,08	0,58	0,12	0,55	0,10	0,48	0,08	0,42	0,17	0,58	0,08
Al2O3	21	0,33	20,64	0,48	20,37	1,27	21,03	0,55	20,99	0,34	21,42	1,35	20,76	0,72
Fe2O3	5	0,87	4,93	0,29	5,18	0,42	4,56	0,61	4,59	0,25	4,57	0,74	5,56	1,72
MnO	0	0,04	0,15	0,04	0,17	0,11	0,14	0,02	0,14	0,05	0,17	0,08	0,11	0,02
MgO	1	0,17	0,69	0,13	0,69	0,26	0,52	0,11	0,49	0,04	0,53	0,10	0,53	0,08
CaO	5	1,09	5,01	0,15	5,33	1,64	4,64	0,78	4,49	0,22	5,22	1,25	4,76	0,46
Na2O	6	1,28	8,30	0,73	7,20	0,62	7,71	1,50	7,97	0,89	6,64	1,01	7,55	0,87
K2O	7	2,29	5,71	0,87	6,50	2,01	7,29	1,94	6,35	0,94	7,15	2,42	6,90	1,68
P2O5	0	0,02	0,22	0,03	0,38	0,14	0,22	0,05	0,17	0,06	0,30	0,38	0,29	0,16
total	100		100		100		100		100		100		100	
F (ppm)	5048	559	5490	1305	5094	977	5675	655	5895	412	5141	832	5477	418
Cl (ppm)	8635	656	8396	1417	8432	1147	9881	1132	9668	714	9110	1545	9487	50
Na2O+K2O	13	1,49	13,80	0,27	13,48	2,14	14,75	0,96	14,08	0,29	13,59	2,05	14,22	0,90
Na/K	2		2,59		2,10		2,00		2,25		1,84		2,02	
AI	0,6		0,7		0,7		0,7		0,7		0,6		0,7	

1822 - 1906 - 1944

1822

Eruptive unit	1822		1822		1822		1822		1822		1822	
Sample	74		1		6		2		7		13	
n		σ (%)		σ (%)		σ (%)		σ (%)		σ (%)		σ (%)
SiO2	47,28	0,48	47,32	0,59	47,44	0,37	46,79	0,81	47,00	0,43	46,96	0,39
TiO2	0,97	0,16	1,11	0,14	1,11	0,15	1,18	0,18	1,06	0,15	1,12	0,17
Al2O3	18,02	0,18	17,85	0,31	17,95	0,28	17,54	1,27	17,45	0,60	17,73	0,59
Fe2O3	11,29	0,51	11,57	0,53	11,54	0,53	11,79	1,00	12,17	0,35	12,24	0,53
MnO	0,19	0,04	0,25	0,05	0,22	0,06	0,21	0,05	0,20	0,03	0,21	0,06
MgO	3,30	0,09	3,33	0,19	3,18	0,09	3,59	0,84	3,52	0,54	3,46	0,32
CaO	9,31	0,23	9,26	0,48	8,73	0,34	9,88	1,59	9,28	0,88	9,30	0,58
Na2O	3,46	0,15	3,98	0,26	4,19	0,29	3,90	0,52	4,00	0,39	3,92	0,32
K2O	5,36	0,23	4,53	0,37	4,90	0,25	4,30	0,71	4,50	0,34	4,24	0,35
P2O5	0,82	0,10	0,79	0,10	0,74	0,10	0,82	0,11	0,81	0,09	0,82	0,14
total												
F (ppm)	3564	631	3683	516	3058	372	3790	886	3610	416	3737	748
Cl (ppm)	7341	166	7612	392	7234	253	7414	1315	7763	361	7783	511
Na2O+K2O	8,82	0,29	8,51	0,53	9,09	0,49	8,20	1,16	8,50	0,66	8,16	0,59
Na/K	1,12	0,06	1,54	0,14	1,49	0,08	1,60	0,19	1,55	0,12	1,61	0,14
AI	0,5		0,5		0,5		0,5		0,5		0,5	

1906

Eruptive unit	1906		1906		1906		1906		1906		1906	
Sample	25		24		6		2		3		5	
n		σ (%)		σ (%)		σ (%)		σ (%)		σ (%)		σ (%)
SiO2	46,69	0,39	46,74	0,33	46,85	0,34	46,92	0,36	46,31	0,69	46,76	0,47
TiO2	1,19	0,15	1,17	0,12	1,19	0,16	1,20	0,16	1,23	0,11	1,13	0,15
Al2O3	17,60	0,29	17,64	0,23	17,49	0,32	17,50	0,53	17,54	0,41	17,58	0,28
Fe2O3	12,29	0,81	11,41	0,57	11,51	0,37	11,82	0,63	11,80	0,71	11,91	0,37
MnO	0,21	0,07	0,22	0,08	0,18	0,04	0,24	0,09	0,17	0,06	0,22	0,04
MgO	3,88	0,24	4,04	0,09	4,08	0,13	4,07	0,57	4,10	0,43	3,95	0,24
CaO	9,45	0,53	9,85	0,19	9,86	0,20	9,48	1,33	9,87	0,73	9,52	0,43
Na2O	4,05	0,53	3,62	0,14	3,58	0,12	4,09	0,70	3,75	0,56	4,00	0,25
K2O	3,63	0,51	4,41	0,20	4,41	0,15	3,63	0,63	4,24	0,67	3,98	0,19
P2O5	1,01	0,12	0,91	0,11	0,87	0,09	1,04	0,09	0,98	0,11	0,95	0,12
total												
F (ppm)	3947	860	3598	419	3333	479	4506	715	3805	569	3884	736
Cl (ppm)	6470	538	6007	234	6001	139	6268	594	6279	833	6334	788
Na2O+K2O	7,68	1,00	8,03	0,31	7,99	0,21	7,72	1,31	8,00	0,64	7,98	0,31
Na/K	1,95	0,15	1,43	0,06	1,41	0,06	1,96	0,09	1,58	0,31	1,75	0,14
AI	0,4		0,5		0,5		0,4		0,5		0,5	

1944

Eruptive unit	A		A		B		B		B		E		E	
Sample	34		9		12		30		7		2		8	
n		σ (%)		σ (%)		σ (%)		σ (%)		σ (%)		σ (%)		σ (%)
SiO2	48,66	0,36	47,78	0,45	48,36	0,42	48,28	0,49	48,05	0,51	48,58	0,44	48,06	1,13
TiO2	1,22	0,11	1,22	0,13	1,10	0,10	1,15	0,10	1,22	0,12	1,20	0,13	1,20	0,15
Al2O3	17,79	0,44	17,57	0,27	17,78	0,19	17,73	0,27	17,49	0,21	17,74	0,35	17,45	1,33
Fe2O3	10,45	0,31	10,10	0,33	9,81	0,34	9,86	0,46	10,10	0,47	9,49	0,58	10,18	0,93
MnO	0,23	0,03	0,20	0,07	0,19	0,08	0,21	0,06	0,20	0,07	0,22	0,08	0,20	0,07
MgO	3,50	0,30	4,00	0,07	3,92	0,11	3,98	0,13	4,17	0,19	3,91	0,24	3,93	1,06
CaO	8,52	0,51	9,70	0,22	9,42	0,26	9,42	0,23	9,94	0,37	9,73	0,47	9,76	2,07
Na2O	4,32	0,23	3,78	0,14	3,56	0,13	3,93	0,21	3,69	0,18	3,97	0,32	4,05	0,70
K2O	4,20	0,28	4,54	0,17	4,78	0,18	4,34	0,19	3,98	0,25	4,02	0,38	3,90	1,02
P2O5	1,09	0,12	1,10	0,10	1,09	0,12	1,11	0,16	1,16	0,13	1,14	0,12	1,28	0,62
total	100		100		100		100		100		100		100	
F (ppm)	3975	447	3124	343	2728	353	3297	290	3311	298	3311	360	3921	1533
Cl (ppm)	5515	404	4952	162	4648	232	5039	165	4981	189	4956	430	4967	760
Na2O+K2O	8,52	0,41	8,33	0,19	8,33	0,25	8,28	0,25	7,67	0,34	7,99	0,63	7,95	1,44
Na/K	1,79		1,45		1,30		1,58		1,62		1,72		1,72	
AI	0,5		0,5		0,5		0,5		0,4		0,5		0,5	

H		H		H	
4		6		8	
	σ (%)		σ (%)		σ (%)
47,76	0,47	48,51	0,33	47,65	0,53
1,25	0,11	1,18	0,12	1,24	0,11
17,55	0,41	17,54	0,38	17,50	0,33
10,35	0,42	10,38	0,45	10,93	0,40
0,21	0,07	0,20	0,08	0,20	0,08
3,86	0,18	3,82	0,13	3,79	0,15
9,65	0,54	9,41	0,29	9,53	0,39
4,05	0,30	4,06	0,37	4,32	0,26
4,14	0,48	3,69	0,21	3,55	0,35
1,19	0,15	1,22	0,14	1,29	0,16
100		100			
3767	409	3745	495	4358	472
4975	278	5007	440	5185	213
8,19	0,62	7,74	0,34	7,88	0,41
1,92		2,14			
0,5		0,4		0,5	

Table S3

Pomice di base

Comment	VPB2 bl Px	VPB2 bl Px	VPB2 gr Px	VPB2 bl Px	VPB2 bl Px	VPB2 gr Px	VPB2 gr Px	VPB2 gr Px	VPB2 bl Px	VPB2 Fd
SiO2	57,71	56,16	56,85	55,98	56,85	55,43	54,32	55,76	55,11	60,24
TiO2	0,18	0,51	0,36	0,36	0,51	0,85	0,56	0,60	0,47	0,26
Al2O3	21,60	22,22	22,37	22,32	22,07	20,09	19,56	19,97	22,04	21,90
Fe2O3	2,98	2,87	2,39	2,62	3,49	5,84	5,58	5,11	3,61	1,09
MnO	0,17	0,11	0,02	0,10	0,11	0,40	0,63	0,49	0,17	0,09
MgO	0,21	0,15	0,10	0,09	0,15	0,25	0,11	0,03	0,05	0,05
CaO	7,08	7,41	7,68	2,93	3,90	5,88	5,90	5,49	3,66	2,14
Na2O	3,18	3,04	3,04	5,01	3,38	3,57	5,97	5,18	6,23	3,69
K2O	6,27	5,74	5,46	10,59	9,43	7,28	7,26	7,24	8,66	10,55
P2O5	0,60	1,79	1,74	0,00	0,13	0,39	0,13	0,13	0,00	0,00
total	100	100	100	100	100	100	100	100	100	100
F ppm	1615	1830	2248	2627	2643	3780	4946	4984	5300	5498
Cl ppm	6973	7299	7020	7468	7270	7094	6917	6891	6809	6497
Na2O+K2O	9,46	8,78	8,50	15,60	12,81	10,85	13,23	12,42	14,89	14,24
Na/K	0,88	0,92	0,97	0,82	0,62	0,85	1,43	1,24	1,25	0,61

**Greenish**	VV3	VV3	VV5	VV5	VV5
	GDPx-MI	PTPx-MI	GDPx-MI	GDPx-MI	PTPx-MI
number	41	45	43	44	46
SiO2	61,02	58,50	59,84	60,42	59,74
TiO2	0,35	0,45	0,27	0,44	0,03
Al2O3	19,49	19,09	19,20	19,21	19,49
FeO	2,32	3,90	2,47	2,97	2,45
MnO	0,14	0,16	0,15	0,14	0,22
MgO	0,19	0,31	0,34	0,23	0,38
CaO	2,51	3,29	2,65	2,86	2,94
Na2O	3,30	4,51	4,05	4,12	2,72
K2O	9,71	8,42	9,40	8,54	10,85
P2O5	0,02	0,09	0,02	0,01	0,08
F	3833	3294	4218	3247	5346
Cl	5830	9363	12089	7513	5678
Total	**100,00**	**100,00**	**100,00**	**100,00**	**100,00**

MERCATO

Comment	VM2 Px g
SiO2	57,61
TiO2	0,10
Al2O3	22,17
Fe2O3	1,95
MnO	0,29
MgO	0,06
CaO	1,47
Na2O	9,82
K2O	6,53
P2O5	0,00
total	100
F ppm	3112
Cl ppm	6412
Na2O+K2O	16
Na/K	2,62

AP1 eruption

Comment	AP1 base Px MI				
SiO2	55,89	56,32	55,42	55,81	56,40
TiO2	0,33	0,40	0,57	0,22	0,30
Al2O3	21,88	21,89	21,91	21,29	22,17
Fe2O3	3,20	2,84	3,71	3,69	2,84
MnO	0,45	0,35	0,62	0,27	0,37
MgO	0,00	0,15	0,07	0,07	0,14
CaO	3,48	4,40	4,10	3,07	2,42
Na2O	6,47	4,46	4,86	5,65	5,54
K2O	8,30	7,96	8,35	9,15	9,81
P2O5	0,00	1,24	0,39	0,77	0,00
total	100	100	100	100	100
F ppm	2778	2737	2099	2686	3690
Cl ppm	6286	6177	5870	6242	4895
Na2O+K2O	15	12	13	15	15
Na/K	1,35	0,97	1,01	1,07	0,98

Pollena

Comment	VP5-GDFDT-MI	VP5-GDFDT-MI	VP5-GDFDT-MI	VP5-GDFDT-MI	VP5-PTFDT-MI	VP5-GDPx-MI	VP5-GDPx-MI	VP5-GDPx-MI
SiO2	55,31	53,88	54,26	55,76	55,71	52,06	51,91	51,28
TiO2	0,06	0,30	0,24	0,14	0,16	0,45	0,32	0,45
Al2O3	23,12	22,45	23,13	22,76	22,84	23,83	23,65	22,90
Fe2O3	2,51	3,54	3,05	2,61	2,50	3,36	3,81	5,01
MnO	0,17	0,20	0,18	0,13	0,19	0,20	0,28	0,21
MgO	0,07	0,12	0,06	0,07	0,07	0,08	0,12	0,36
CaO	2,18	3,64	2,40	2,11	2,16	2,63	2,74	4,01
Na2O	6,96	7,15	7,43	7,00	6,68	8,17	8,20	7,33
K2O	9,62	8,71	9,15	9,42	9,68	9,09	8,97	8,41
P2O5	0,01	0,01	0,11	0,01	0,01	0,12	0,00	0,03
total	100,00	100,00	100,00	100,00	100,00	100,00	100,00	100,00
F ppm	3560	3982	3643	3372	3463	4820	4802	4384
Cl ppm	6762	8302	6654	6489	6192	9047	8335	8367
Na2O+K2O	16,58	15,86	16,58	16,41	16,36	17,26	17,17	15,75
Na/K	1,26	1,43	1,41	1,29	1,20	1,56	1,59	1,52

Comment	VP5-GDPx-MI	VP8-GDFDT-MI	VP8-PTFDT-MI	VP8-PTFDT-MI	VP8-GDPx-MI	VP8-GDPx-MI
SiO_2	51,43	56,20	56,37	56,61	51,00	50,55
TiO_2	0,02	0,16	0,09	0,04	0,40	0,55
Al_2O_3	24,26	22,41	22,69	22,71	23,19	22,49
Fe_2O_3	3,70	2,11	2,04	2,20	4,67	5,40
MnO	0,16	0,14	0,13	0,12	0,17	0,16
MgO	0,13	0,07	0,07	0,03	0,22	0,50
CaO	3,80	1,98	1,88	1,95	4,65	5,61
Na_2O	6,88	7,12	6,88	6,44	7,39	6,89
K_2O	9,52	9,80	9,85	9,88	8,26	7,77
P_2O_5	0,08	0,00	0,00	0,03	0,04	0,07
total	100,00	100,00	100,00	100,00	100,00	100,00
F ppm	3882	3026	2983	2964	4054	3947
Cl ppm	7910	5600	5573	5459	7900	7406
Na_2O+K_2O	16,41	16,92	16,73	16,31	15,66	14,66
Na/K	1,26	1,26	1,21	1,13	1,56	1,54

	**1631**								
	1631SL-B	Px 500							
	c3-MI2b	c3-MI2c	c3-MI4	c3-MI4b	c4-MI5a	c4-MI5b	c4-MI6	c4-Px	c6-MI10
SiO2	51,83	57,03	56,88	54,38	58,34	57,97	55,75	53,88	59,99
TiO2	0,83	0,97	0,38	0,43	0,40	0,41	0,59	0,55	0,24
Al2O3	17,76	23,48	23,37	23,24	24,73	24,66	23,81	20,94	24,81
Fe2O3	6,45	4,54	5,10	4,31	3,91	4,06	5,06	6,16	4,02
MnO	0,11	0,21	0,10	0,14	0,15	0,18	0,19	0,29	0,11
MgO	4,29	0,47	0,37	0,35	0,21	0,25	1,00	2,76	0,36
CaO	12,23	4,87	5,36	4,74	4,41	4,49	5,68	8,42	5,04
Na2O	5,55	9,63	7,10	6,72	4,41	4,46	4,15	3,16	3,24
K2O	3,90	6,12	6,93	6,18	11,89	12,16	10,81	8,41	10,52
P2O5	0,12	0,14	0,29	0,14	0,15	0,17	0,22	0,90	0,18
total	100,00	100,00	100,00	100,00	100,00	100,00	100,00	100,00	100,00
F ppm									
Cl ppm	6130	8600	9310	9490	8420	8230	7790	5880	8020

	c11-MI16	c11-MI16	c11-MI16b	c11-MI16c	c11-MI16d	c11-M17	c11-Px	c8-MI20a	c17-MI26
SiO2	55,84	59,43	57,82	58,59	58,53	56,05	55,48	57,18	59,03
TiO2	0,36	0,22	0,17	0,37	0,26	0,54	0,70	0,39	0,84
Al2O3	22,70	23,93	23,57	23,67	23,35	22,86	22,89	23,61	24,20
Fe2O3	3,94	3,15	3,07	3,15	3,08	4,65	4,41	4,08	4,33
MnO	0,22	0,18	0,19	0,06	0,00	0,12	0,16	0,19	0,07
MgO	0,49	0,49	0,33	0,55	0,52	0,31	0,39	0,48	0,69
CaO	4,18	3,40	3,06	3,46	3,43	5,87	6,05	3,94	6,31
Na2O	3,70	5,65	5,46	5,85	5,42	4,62	5,08	4,85	4,15
K2O	11,61	11,04	11,15	10,87	11,23	11,00	10,32	10,41	9,28
P2O5	0,12	0,10	0,15	0,02	0,08	0,19	0,10	0,40	0,57
total	100,00	100,00	100,00	100,00	100,01	100,00	100,00	100,00	100,00
F ppm					1640	1660			
Cl ppm	7750	7680	8570	6750	7930	10630	9750	9240	9070

	c17-MI26b	c17-MI26c	c17-MI26d	c17-MI28a	c17-MI28b	c23-MI30a	c23-MI30b	c24-MI31	c24-MI31a
SiO2	59,73	59,10	60,19	58,72	58,11	58,52	55,89	55,87	58,86
TiO2	0,81	0,52	0,53	0,43	0,61	0,63	0,91	0,37	0,33
Al2O3	24,15	24,80	25,14	24,07	24,11	24,05	23,39	23,46	24,07
Fe2O3	3,41	3,48	3,94	3,45	3,55	4,86	5,01	4,40	3,47
MnO	0,20	0,02	0,14	0,15	0,30	0,05	0,08	0,09	0,09
MgO	0,53	0,47	0,47	0,42	0,39	0,26	0,27	0,38	0,22
CaO	6,20	5,52	5,78	5,43	5,40	5,97	5,87	4,39	3,20
Na2O	4,44	4,39	3,29	4,50	4,63	1,71	2,17	4,85	5,37
K2O	9,44	9,77	9,36	9,89	9,21	9,15	8,87	10,30	11,87
P2O5	0,51	0,61	0,60	0,50	0,52	0,42	0,36	0,11	0,09
total	100,00	100,00	100,00	100,00	100,00	100,00	100,00	100,00	100,00
F ppm									
Cl ppm	8050	7330	9730	8960	8890	9980	8620	7790	7300

	c24-MI31b	c25-MI34a	c25-MI34b	c25-MI35	c25-MI36a	c25-MI36b	c25-MI37a	c25-MI37b	c26-MI38
SiO2	58,53	55,88	56,33	55,25	57,31	55,98	56,74	55,86	56,80
TiO2	0,32	0,42	0,35	0,51	0,60	0,48	0,30	0,48	0,36
Al2O3	23,92	22,63	22,26	22,09	22,73	22,46	22,73	22,46	23,29
Fe2O3	3,08	4,68	3,96	4,15	4,34	4,36	4,79	4,69	3,82
MnO	0,00	0,06	0,15	0,12	0,14	0,23	0,04	0,16	0,15
MgO	0,49	0,47	0,46	0,68	0,35	0,26	0,41	0,51	0,29
CaO	4,41	5,13	5,49	5,23	5,60	5,39	5,09	5,08	5,64
Na2O	5,55	5,27	5,30	4,88	4,84	5,15	5,29	5,11	5,28
K2O	11,80	10,38	10,53	10,40	11,07	10,65	11,09	10,71	10,98
P2O5	0,05	0,13	0,15	0,14	0,12	0,15	0,18	0,13	0,14
total	100,03	100,00	100,00	100,00	100,00	100,00	100,00	100,00	100,00
F ppm	980								
Cl ppm	7100	9360	10330	9180	9200	9910	8200	10210	9730

	c26-39a	c26-39b	c26-MI40a	c26-MI40b	c26-MI40c	c26-MI40d	c26-MI41a	c26-MI41b	c26-MI41c	c27-MI42
SiO2	55,62	60,74	55,16	56,06	54,98	55,93	55,80	54,61	54,80	57,19
TiO2	0,61	0,67	0,44	0,44	0,33	0,66	0,40	0,52	0,49	0,60
Al2O3	23,14	23,24	22,19	23,08	22,61	22,69	22,45	22,12	22,59	24,43
Fe2O3	3,94	5,06	4,67	4,62	4,71	4,62	4,37	4,66	4,73	3,90
MnO	0,15	0,10	0,24	0,09	0,06	0,08	0,13	0,15	0,11	0,24
MgO	0,31	0,38	0,45	0,40	0,43	0,42	0,34	0,37	0,48	0,17
CaO	5,56	6,84	6,12	5,95	5,99	5,97	5,87	6,01	5,67	3,72
Na2O	5,21	6,06	5,10	4,80	4,91	4,51	5,22	5,05	5,11	4,60
K2O	11,15	13,69	10,68	10,97	10,56	10,75	10,65	10,37	10,52	11,44
P2O5	0,11	0,24	0,10	0,09	0,21	0,17	0,09	0,20	0,11	0,35
total	100,00	100,00	100,00	100,00	100,00	100,00	100,00	100,00	100,00	100,00
F ppm										
Cl ppm	9330	9880	10620	9500	9570	10120	9920	9650	9580	9910

Supplementary Information S4: The evolution through time of the shallow plumbing system for the last 22 ky period of activity of Mount Somma – Vesuvius.

A. **The pressure is only deduced from the choice of a Cl experimental solubility law.** The uncertainty is within symbol size excepted for the Greenish eruption and the three last eruptions (1822, 1906, 1944) for which a bow corresponds to the domain of pressure deuced from experimental Cl solubility.

B. **The pressure is only deduced from the Cl solubility modelling using the model developed by Webster and collaborators.** The uncertainty is within symbol size.

The figure 8 is the manuscript corresponds to the best pressure estimate as discussed within the text. When no Cl experimental solubility law exists, the pressure is less constrained than the pressure estimated by the Cl solubility modelling that takes into account the Cl solubility dependence with the melt composition.

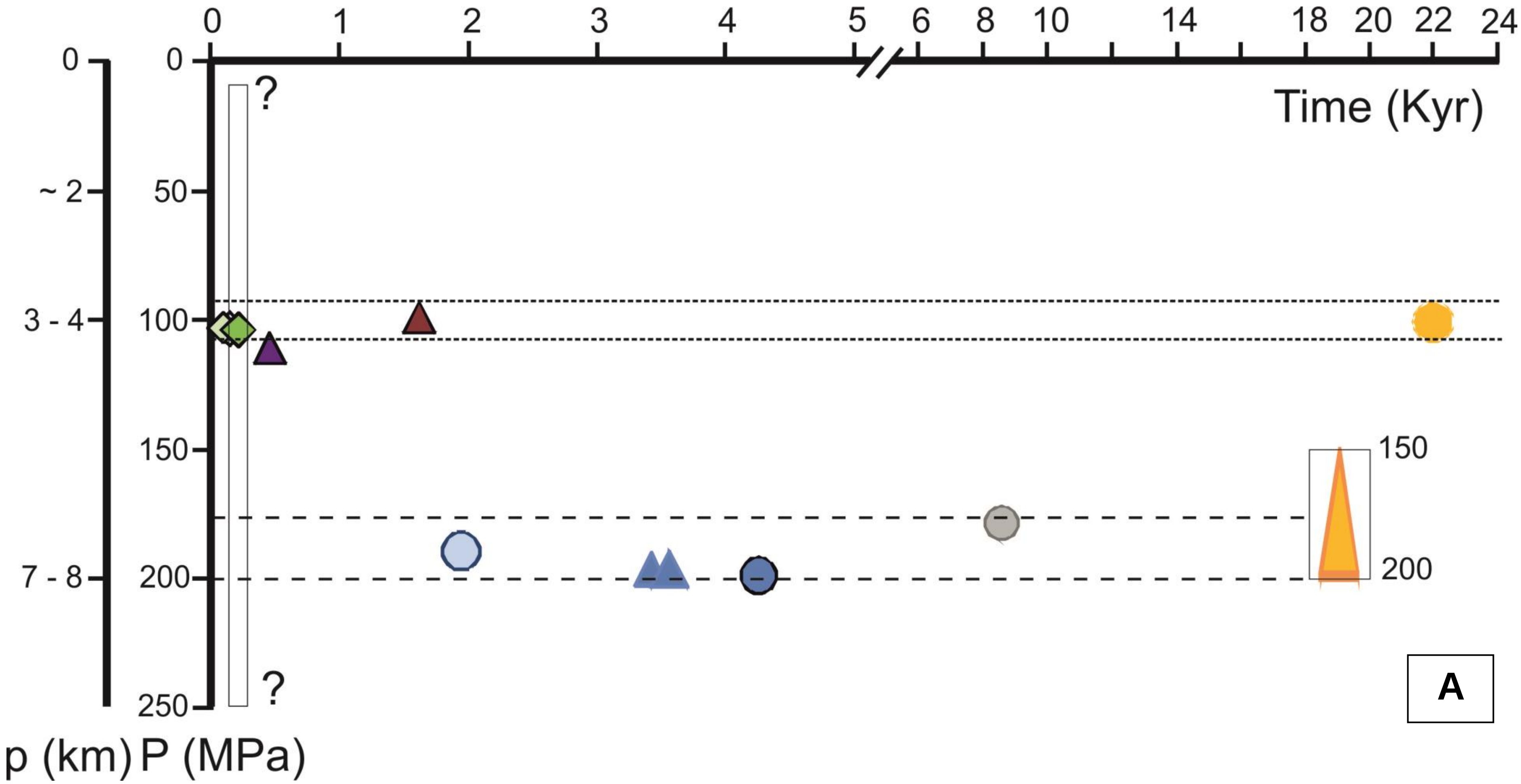

0
1
2
3
4
5
6
8
10
14
18
20
22
24
Time (Kyr)
0
~ 2
3 - 4
7 - 8
0
50
100
150
200
250
?
?
150
200
A
p (km)
P (MPa)

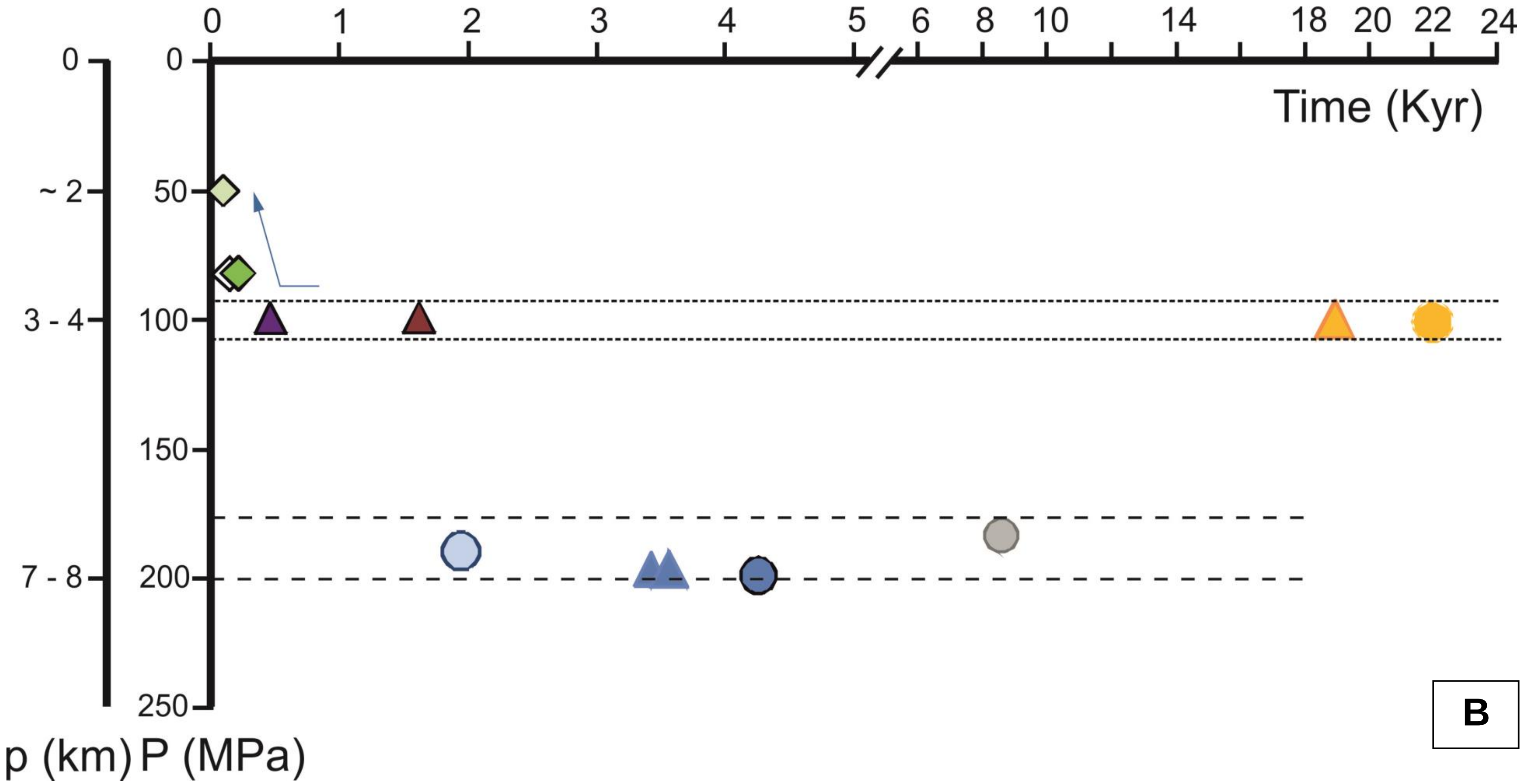

0
1
2
3
4
5
6
8
10
14
18
20
22
24
Time (Kyr)
0
~ 2
3 - 4
7 - 8
0
50
100
150
200
250
B
p (km)
P (MPa)
